# The Excised Super-thin Skin as a Flap Sizer for Finger and Hand Free-Mini-Flap Reconstruction

**DOI:** 10.1097/GOX.0000000000001767

**Published:** 2018-04-13

**Authors:** Ryo Karakawa, Hidehiko Yoshimatsu

**Affiliations:** From the Department of Plastic and Reconstructive Surgery, The University of Tokyo, Tokyo, Japan.

In the setting of finger and hand free-flap reconstruction, it is difficult to revise the transferred flap if it is too bulky or too small. To prevent secondary revision, a flap that is of the same size as the defect should be transferred. Usually, the flap is designed by measuring the size of the defect with a ruler. However, it is difficult to design a flap that perfectly fits the defect using this method because the thickness, flexibility, and texture of the flap are not taken into consideration. To address this challenge, we used an excised skin as a flap sizer to design a flap that perfectly fits the defect.

**Fig. 1.**
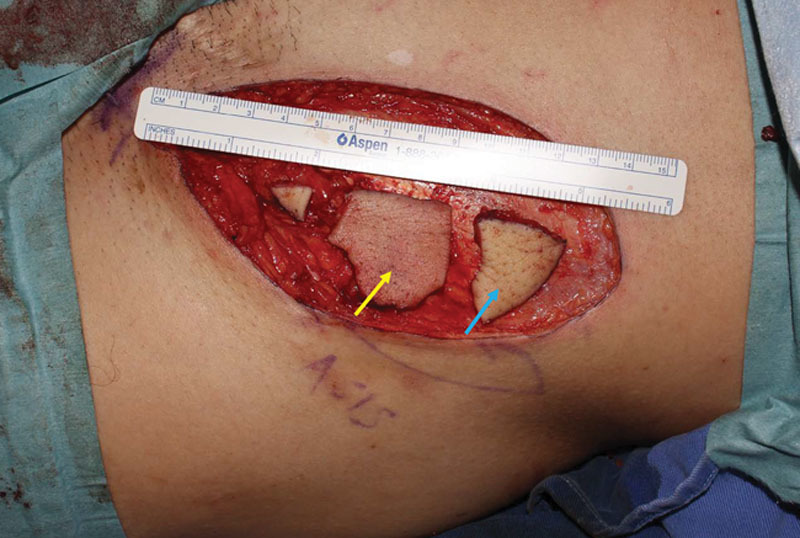
The SCIP flap was divided into 2 pieces: the flap with pedicle (yellow arrow) and the excised skin that was to be used as the flap sizer (blue arrow).

**Fig. 2.**
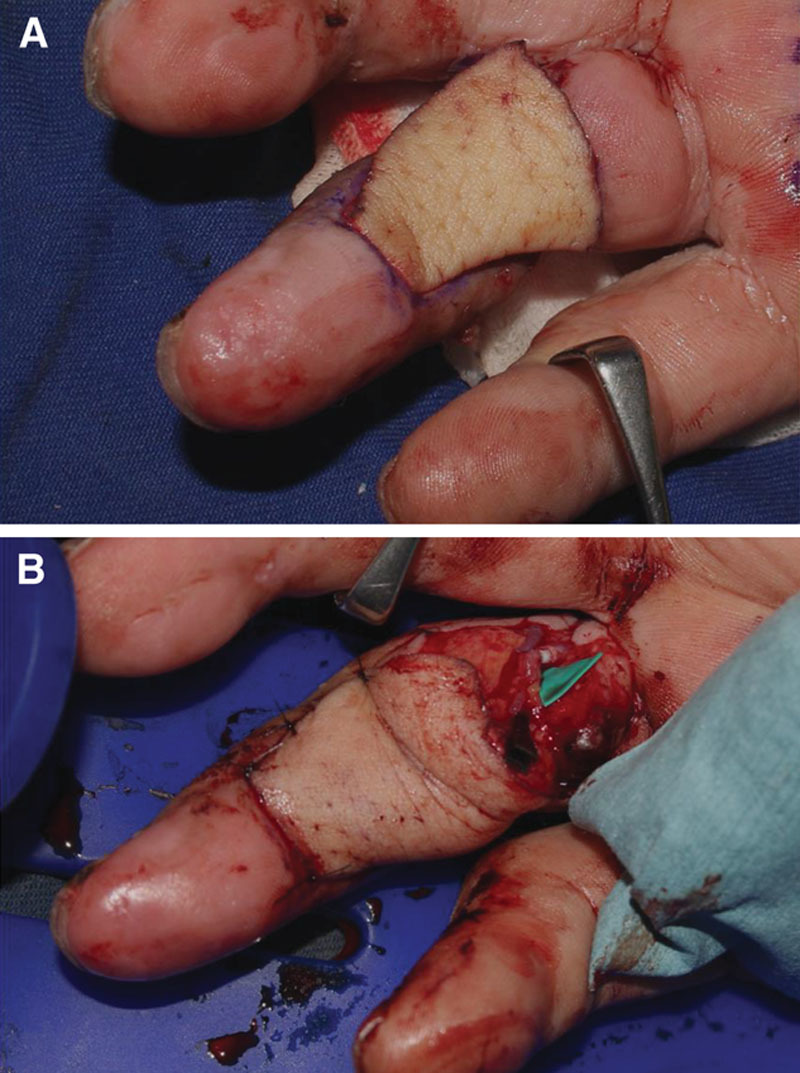
A, The flap sizer was processed to the size of the defect. B, The SCIP mini flap was adjusted to the size of the flap sizer and transferred.

Finger reconstruction using a super-thin mini superficial circumflex iliac perforator (SCIP) flap was planned for an injured patient.^1^ A 3 × 8 cm spindle-shaped SCIP flap was designed and elevated at the superficial fascial plane.^2^ The flap was then thinned, maintaining the blood supply^1^ and divided into 2 pieces: the flap with pedicle and the excised skin that was to be used as the flap sizer (Fig. 1). The excised skin was processed to the size of the defect. The SCIP mini flap was then adjusted to the size of the flap sizer (Fig. 2). The digital artery of the ring finger was anastomosed to the branch of the superficial circumflex iliac artery and the dorsal vein of the ring finger was anastomosed to the superficial circumflex iliac vein.

Using the excised skin as a flap sizer has 3 main advantages. First, the use of an excised skin flap sizer that has the same thickness, flexibility, and texture as the flap helps to design a flap that properly fits the defect. Furthermore, considering the “aesthetic unit” of the finger, aesthetically and functionally satisfactory results can be obtained.^3^ Second, because it is possible to adjust the size of the flap before cutting off the pedicle, the ischemic time of the flap will be shortened. Third, in this method, there is no unnecessary excision of intact skin because, in either case, the additional excision of intact skin is needed for the closure of donor site.

This article highlights the possibility of using excised super-thin skin as a flap sizer for finger and hand free-mini-flap reconstruction. Although further clinical investigation will be required to confirm its efficacy, this method allows for aesthetically and functionally satisfactory results to be obtained.
